# A video based feedback system for control of an active commutator during behavioral physiology

**DOI:** 10.1186/s13041-015-0152-8

**Published:** 2015-10-12

**Authors:** Mootaek Roh, Thomas J. McHugh, Kyungmin Lee

**Affiliations:** Department of Anatomy, Brain Science & Engineering Institute, Behavioral Neural Circuitry and Physiology Laboratory, Kyungpook National University Graduate School of Medicine, 2-101, Dongin-dong, Jung-gu, Daegu, 700-842 South Korea; Laboratory for Circuit and Behavioral Physiology, RIKEN Brain Science Institute, 2-1 Hirosawa, Wako-shi, Saitama, 351-0198 Japan

**Keywords:** *In vivo*, Mice, Motorized commutator, Video tracking

## Abstract

**Background:**

To investigate the relationship between neural function and behavior it is necessary to record neuronal activity in the brains of freely behaving animals, a technique that typically involves tethering to a data acquisition system. Optimally this approach allows animals to behave without any interference of movement or task performance. Currently many laboratories in the cognitive and behavioral neuroscience fields employ commercial motorized commutator systems using torque sensors to detect tether movement induced by the trajectory behaviors of animals.

**Results:**

In this study we describe a novel motorized commutator system which is automatically controlled by video tracking. To obtain accurate head direction data two light emitting diodes were used and video image noise was minimized by physical light source manipulation. The system calculates the rotation of the animal across a single trial by processing head direction data and the software, which calibrates the motor rotation angle, subsequently generates voltage pulses to actively untwist the tether. This system successfully provides a tether twist-free environment for animals performing behavioral tasks and simultaneous neural activity recording.

**Conclusions:**

To the best of our knowledge, it is the first to utilize video tracking generated head direction to detect tether twisting and compensate with a motorized commutator system. Our automatic commutator control system promises an affordable and accessible method to improve behavioral neurophysiology experiments, particularly in mice.

**Electronic supplementary material:**

The online version of this article (doi:10.1186/s13041-015-0152-8) contains supplementary material, which is available to authorized users.

## Background

To study neural function and its behavioral relevance in cognitive and behavioral neuroscience fields it is crucial to record neuronal activity in freely behaving animals [1–3]. Recent advances in wireless recording technology have provided unprecedented freedom to animals performing behavioral tasks while simultaneously recording neural activity [4]. However, the limited number of recording channels and the weight of battery the animal must carry restrict the use of wireless systems, especially in mice. Thus, most laboratories employ a conventional recording system which uses a tether cable connecting a headstage and an amplifier, however, the tether cable is often rendered twisted during animal’s behavior, limiting motion and preventing free movement. The typical solution is to employ a commutator, a device that prevent tether from twisting, allowing animals to move with little tether interferences. An intrinsic drawback of the commutator is that the amount of torque required for passive rotation increases proportionally to the number of circuits (i.e., channels) it possesses. A three-channel commutator system requiring little torque for rotation was designed without a motorized system [5], however, motorized commutator systems are preferred for multichannel *in vivo* recordings in rodents. Though a motorized commutator based on a hall-effect sensor was previously developed [6], most motorized commutators commercially available have limitations, including expense, durability, sensitivity and adaptability to small rodents such as mice. In this study, we describe a novel and simpler approach to automatically motorize a commutator control system using video tracking information in lieu of a torque sensor. The animal’s head position and direction are extracted from two head mounted LEDs’ in the video image. To improve the signal-to-noise ratio (SNR) of the parameters, the focus level of LEDs and camera were manipulated. The trace of head direction is sampled during a single trial of behavioral task and subsequently analyzed by a custom algorithm which then triggers a calibrated DC motor to correct any twist in the tether. This technique was applied to mice performing a behavioral task and allowed successful performance without tether interference.

## Results

### Strategy for develop a track-based commutator system

**Fig. 1:**
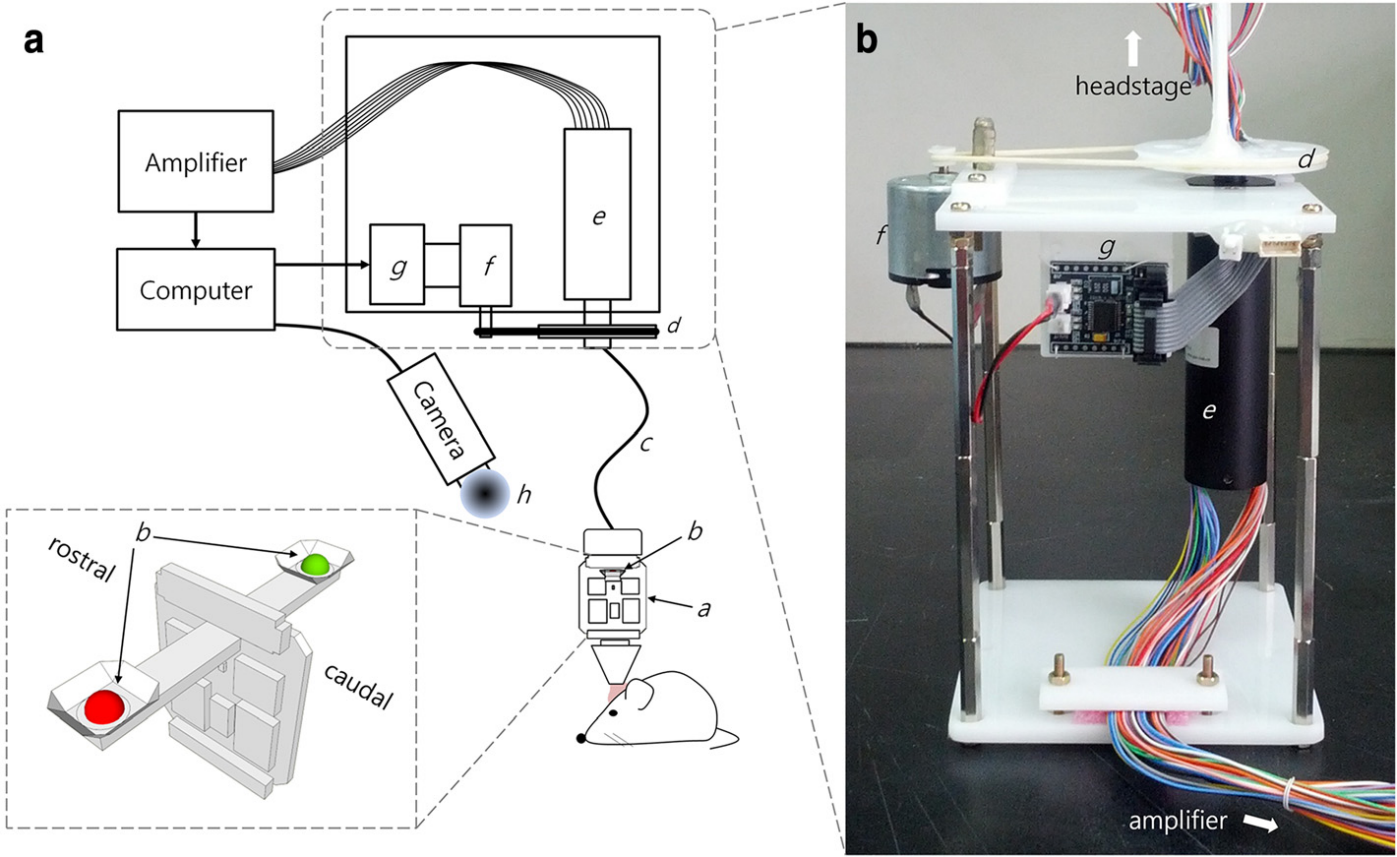
Designed commutator control system. **a** Schematic diagram of the system. Detailed design of LED surrounding reflectors are shown in inset. **b** Design of constructed motorized commutator. *a*: headstage, *b*: LEDs, *c*: tether, *d*: pulley, *e*: slip-ring, *f*: DC motor, *g*: motor control board, *h*: out-of-focused lens

**Fig. 2:**
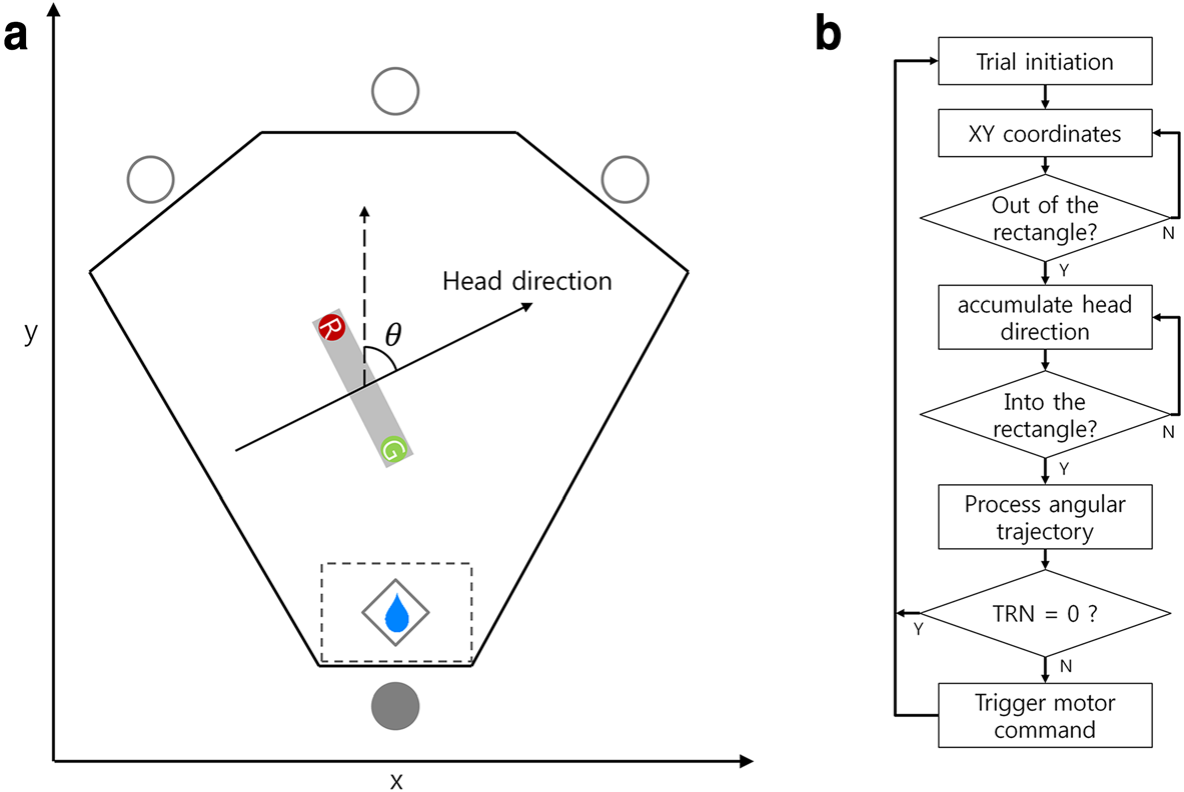
Behavioral task chamber and parameter definitions used for commutator control algorithm. **a** The head position is defined by the mid-point between LEDs, and the head direction is defined by the angle between positive y axis of the video image and rostro-caudal axis of the animal. The underlying polygon indicates the shape of the behavioral chamber. Open circles indicate the positions of visual cues. The diamond and filled circle indicate reward and start nosepoke hole position, respectively. R and G indicate red and green LEDs, respectively. Dashed line indicates a virtual rectangle of the starting position. **b** The flowchart shows how the commutator control algorithm works. After trial initiation, the instantaneous head direction is acquired as soon as the mouse leaves the starting position. When the mouse comes back into the starting position, the trajectory is analyzed to generate total rotation number (TRN) which is used to actuate subsequent motor drive. For more details please see Methods

The commutator system we designed allows for active correction of tether-twisting as a result of behavior via feedback from a video-tracking system. As diagramed in Fig. 1a, the system allows for 36-channel recording and is compatible with the Neuralynx (Bozemon, MT) data acquisition system. We will describe below the four major components of the implementation of this approach: 1) the refinement of the video tracking to improve the accuracy of the acquired data, 2) the mechanical assembly of the commutator system, 3) the development of the algorithm to link the tracking data to the motion of the motor and 4) the calibration of the commutator with open loop tracking. Finally, we report its successful implementation in a modified version of the 5-choice serial reaction time test (Fig. 2).

### Image noise minimization

To improve the SNR of the video images we first manipulated the tracking LEDs mounted on the mouse’s head. Since it is impossible to control the intensity of the commercial LEDs we covered them physically with layers of paraffin film to attenuate the center points’ intensities to prevent saturation. Although we used a narrow 2 mm diameter tether (TETH-HS-36-Litz, Neuralynx) the tether occasionally occluded the LEDs, thus we added curved aluminum reflectors behind the LEDs to widen their spatial coverage (Fig. 1a inset). The position of LEDs were adjusted to be perpendicular to animal’s rostro-caudal axis, that is, red and green LEDs were positioned left and right side of the animal’s head, respectively (Fig. 1a inset). This allowed continuous tracking even when an animal changes its head direction vertically, for example, during grooming or rearing against a wall.

**Fig. 3:**
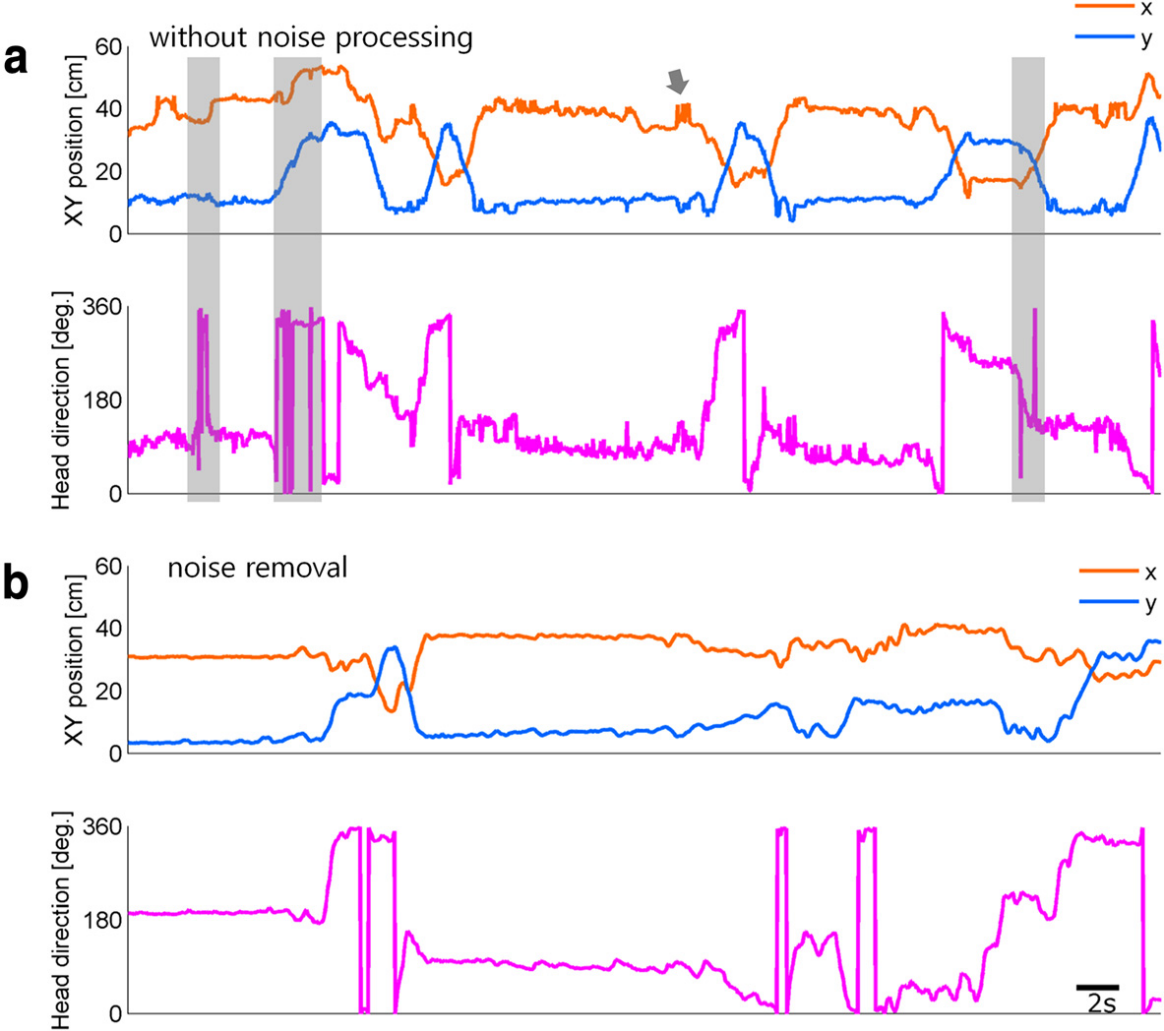
Improvement of signal-to-noise ratio for head position and direction in task-performing mice. **a** Head position and direction without noise processing show abrupt angular change when there are no positional variation (shaded). Detected position is unstable and bouncing as indicated by an arrow. **b** These noises are removed by LED manipulation and image smoothing (see Section Image noise minimization for more details). Abrupt changes are not observed in both X and Y positions. Head directions are smoother than before even though they are not processed offline, such as, digital filtering

Next, as the LEDs could generate spurious reflective signals from the walls of the behavioral chamber the walls were matted via abrasion. In the altered chamber the reflected LED light was diffuse and its intensity was attenuated. Then, to achieve improvement of SNR in video images, incoming images were smoothed at the initial sensing stage (Fig. 1 a.*h*) by adjusting the focus level of camera manually to be slightly out of focus to get pre-smoothing filtered images (Additional file 1: Movie 1). Before noise processing both head position and direction parameters were erratic with bursts of noise (Fig. 3a; shaded area), however, following the SNR improvements described these errors were rarely detected. Head positions were stabilized, indicating that the direction maintains its angle steadily (Fig. 3b). Therefore, this strategy was appropriate in improving the detectability of head position and direction from incoming video images.

### Mechanical assembly of the commutator

We attached a 56 circuit slip-ring capsule (PSR-C56, Pan-Link technology; Fig. 1b.*e*) to connectors (MDR connector, 50 pin, 3 M Korea) compatible with the existing amplifier system (ADPT-HS-36-DRS, Neuralynx). To drive rotation of the commutator a DC motor (5 V FM1502, D&J WITH, Korea; Fig. 1b.*f*) with a small pulley attached to the motor shaft was coupled to a larger pulley glued to rotary joint which connected to the tether (Fig. 1 b.*d*). The commercially available DC motor driver was designed based on TB6612FNG chip (Toshiba; Fig. 1b.*g*) providing bidirectional control (clock- and counter clock-wise). Next we connected the output terminal of the digital IO device (NI PCI-6601, National Instruments) to the motor controller in order to operate the DC motor (Fig. 1a).

### Development of algorithm

The mouse’s head position was defined as the mid-point between the two LEDs and the head direction was defined by the angle composed of a reference line (e. g., y axis of an image) and the midline of animal (rostral to caudal), which is orthogonal to the axis passing both LEDs in the video image (Fig. 2a). The tracking parameters were provided by the data acquisition software, Cheetah (Neuralynx) and customized software was designed using Labview (National Instruments) to apply the algorithm shown in Fig. 2b.

### DC motor calibration

**Fig. 4:**
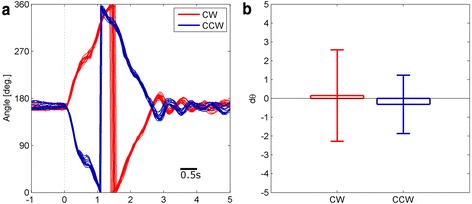
Calibration of DC motor rotation. Single rotation (**a**) Trajectory segments of both CW and CCW direction are aligned to the onset of motor drive (*n* = 15 for CW, *n* = 16 for CCW). Single rotation takes approximately 2.6 s. After rotation, angular positions approach to pre-rotation level. **b** The mean angular difference of pre (−165 to 0 ms) and post (3.7 to 4.7 s) is depicted. Both CW and CCW direction shows nearly zero, indicating no significant angular change after rotation. The error bar indicates mean ± s.d

Motor calibration was performed for precise angular control over repetitive rotations. Figure 4a shows angular position trajectories in both CW and CCW rotation. After confirming that given motor command generates single rotation, repetitive CW- and CCW-rotation was applied in a randomly mixed sequence. Then the segments of each CW and CCW-rotation trajectories were cut and aligned as shown in Fig. 4a. The trajectories of both CW (*n* = 15) and CCW (*n* = 16) show no significant change of direction after motor rotation (Fig. 4b), indicating that DC motor is calibrated to rotate close to 360°.

### Practical application

**Fig. 5:**
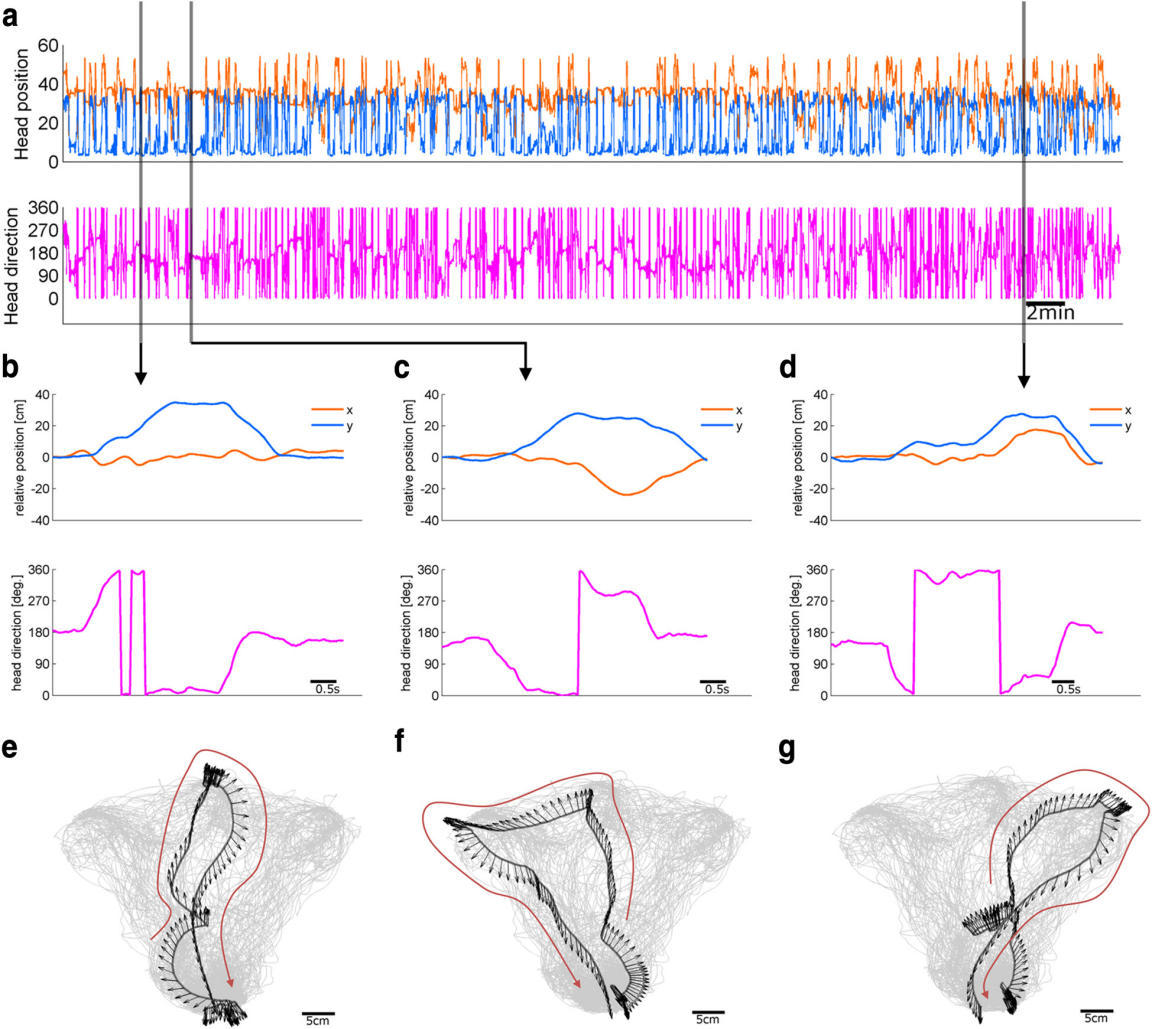
Application of commutator system to task-performing mouse. **a** A whole single session traces of head position and direction, lasting about 55 min. Shaded areas indicate examples shown below. **b**–**d** An example of traces during a trial that the TRN is +1 (**b**), −1 (**c**), and zero (**d**). Each of the position and head direction is depicted in top and bottom panel, respectively. **e**–**g** Actual mouse movement traces during the trials corresponding to (**b**–**d**). Instantaneous head directions are marked as arrows at the position

The designed commutator was finally applied to animals performing behavioral tasks (Additional file 2: Movie 2). The head position and direction of single session, during which mice traveled on avaerge 55 m, are shown in Fig. 5a. During the session, we chose representative CW and CCW trials (Fig. 5b and c), and the corresponding actual movement traces are depicted upon the total traces of the session, as shown in Fig. 5e and f. In Fig. 5e, the mouse nosepokes to start a trial and turns right and moves toward center cue and turns right to come to the reward position. In this case, the TRN is +1, indicating single rotation along CW direction. On the other hand, in Fig. 5f shows CCW rotation, that is the TRN of −1. Figure 5d and g shows a trial that the mouse does not rotate. In Fig. 5g, the mouse nosepokes at the starting position and turns left and, after hesitation, moves toward the right cue. Then it turns right and comes to reward position, indicating half-rotated CW and CCW are canceled out. The resulting TRN is, therefore, zero.

## Discussion

Here we describe a novel motorized commutator system which is automatically controlled by video tracking. The system computed TRN values from low-noise video images and generated voltage pulses to control motor output. A single calibration procedure, performed prior to the start of the experiments, was sufficient to determine how many pulses occupy a single rotation of pulley. The main advantage of this system is its low cost compared to commercial systems and a simple to use software-based calibration which eliminates the need for traditional torque-sensor calibration.

To obtain accurate head direction data using LEDs, video noise (artifact) needs to be removed or minimized. We adopted physical manipulation of light sources (LED) combined with defocused video recording (analog filtering). The noise reduction strategy we describe may be applied to general video tracking to improve raw data quality of head position and direction irrespective of the use of a wired or wireless recording system [1–3].

However, there will be additional modification required to improve the performance of the commutator and to make it into an independent module that can be flexibly integrated into any neural recording system. Currently the commutator uses a commercial neural recording system equipped with video tracking to generate head direction values. In the future, single board computers providing internal program customization and digital output ports may be integrated in this system to remove this dependency [7]. Moreover, since this system uses defocused camera input for noise reduction, another monitoring video is needed for general monitoring purpose. If incoming video streams can be filtered online by digital image processing, it is possible to use a single camera for simultaneous animal monitoring and commutator controls.

Commercial DC motors are cheap and easy to drive, but difficult to precisely control angular positions. Other types of advanced motor systems, such as stepper motors and drivers, may be used to achieve exact angular control without requiring any calibration procedures [8].

In the behavioral task used in this study, animals are required to move specific location of the chamber to initiate, choose, and receive a reward. The rotation counting algorithm was designed based on this behavioral task. Animals spend most of time consuming reward, so a virtual rectangular box around the reward port was set and tether is untwisted as soon as animals enters the box. This, however, can be a problem when animals spend more time outside the box while rotating toward a single direction. Although animals seem insusceptible to a single twist of tether, continuous control of commutator is desirable. To become an independent modular system, this feature will be necessary. Moreover, the system may be further improved in combination with tagging-free video tracking techniques by eliminating light sources on the headstage and adopting computerized video analysis or tracking techniques using accelerometers [9–11].

## Conclusions

In this study, we developed a motorized commutator system which is automatically controlled by video feedback. Previously developed motorized commutator systems employ torque sensors to detect tether twist, which can be effective but faces limitations, especially in smaller rodents. Here we used head directions computed by video tracker system to detect tether twist and use this information to correct the tether position on a trial-by-trial basis. This system successfully provided tether twist-free environment for animals performing our behavioral task during acquisition of neural activity data.

## Methods

### Subject, surgery, and behavioral task

#### Subject

Male C57BL6/J mice (*n* = 2) weighing 20 ~ 25 g were used in this study. Mice were housed individually in a Plexiglas cage under 12 h/12 h light–dark cycle. Water was limited but food was available ad libitum and body weights were maintained about 80 % of free-feeding weight. All procedures regarding animal care and handling were approved by the Institution of Animal Care and Use Committee of Kyungpook national university (South Korea) and all experimental protocols were performed in accordance with the guidelines for the Care and Use of Laboratory Animals of the National Institutes of Health (USA).

#### Surgery

The mice were anesthetized with an intraperitoneal injection of tribromoethanol (Avertin, 0.0125 mg/g of body weight). Deep anesthesia was confirmed by tail and paw pinches, which resulted in no withdrawal behavior. Then mouse head was fixed on a stereotaxic apparatus. Ophthalmic ointment was applied to prevent from drying eyes. After shaving and midline incision, skull surface was cleared using saline. Periosteum was scrapped off and holes were drilled and screws were implanted. A microdrive having 8 tetrodes was implanted above the M1 cortex targeting striatum, and dental cement was applied. After 1 week of recovery period, mouse was introduced to behavioral task.

#### Behavioral task

The schematic drawing of behavioral chamber, which is a modified version used for 5-choice serial reaction time task, is depicted in Fig. 2a [12]. At the start position, mouse was required to nose-poke to initiate a trial. Upon initiation visual cues on the opposite end of the maze were illuminated and the mouse was trained to move toward the cue position and nose-poke to the cued hole to receive reward. Correct nose pokes resulted in water delivery as a reward, but incorrect nose poke resulted in sudden black out of 5 to 8 s as a penalty. Since the water nozzle was located near the start position, mouse had to turn and move back to the start position to consume reward.

### Development of automatic commutator control system

- First step: refinement of the video tracking to improve the accuracy to detect behavioral parameters such as X, Y coordinates or head-directions
- Second step: mechanical assembly of commutator system
- Third step: development of algorithm to compute and judge whether or not the animal has made a turn
- Final step: calibration of commutator with open loop tracking

#### First step: video tracking refinement

- LED manipulation for video noise reduction
  For video tracking, we used digital amplifier (Digital Lynx SX, Neuralynx) with a headstage with two LEDs, red and green mounted alongside (HS-36, Neuralynx). Since it is impossible to control the intensity of the commercial LEDs, we covered the LEDs physically with layers of paraffin films to attenuate the center points’ intensities of LEDs to prevent saturation. Although we used a 2 mm diameter tether (TETH-HS-36-Litz, Neuralynx), sometimes the tether positioned between LEDs and camera, obstructing LED detection. We therefore added the curved aluminum reflectors behind LEDs to widen the spatial coverages of LEDs (Fig. 1a inset).
  The position of LEDs were adjusted to be perpendicular to animal’s rostro-caudal axis, that is, red and green LEDs were positioned left and right side of the animal’s head, respectively (Fig. 1a inset). This allowed continuous tracking even when an animal changes its head direction vertically, for example, during grooming or rearing against wall.
- Improving SNR by image smoothing
  Since the behavioral chamber had glossy walls which could reflect the LEDs, causing errors in detection, the walls were matted via abrasion. In the altered chamber the reflected LED light was diffuse and its intensity was attenuated. To improve the SNR of video image, we smoothed incoming images at the initial sensing stage (Fig. 1a.*h*). The camera provided a dial to control the focusing level manually, so we adjusted the level slightly out of focus to get pre-smoothing filtered images. For general behavioral monitoring purposes, we used an additional video camera.

#### Second step: mechanical assembly

We used 56 circuits slip-ring (PSR-C56, Pan-Link technology; Fig. 1b.*e*). The wires of both ends were soldered to connectors (MDR connector, 50 pin, 3 M Korea) which matches to the adapter of existing amplifier system (ADPT-HS-36-DRS, Neuralynx).

To rotate it using a DC motor (5 V FM1502, D&J WITH, Korea; Fig. 1b.*f*), a pulley of diameter 60 mm was glued to rotary joint which connected to tether (Fig. 1b.*d*). A small sized pulley (diameter of 6 mm) was attached to the DC motor shaft. Those two pulleys were linked using a rubber belt so that motor power was transferred through the pulley. The distance between center points of each pulley was approximately 85 mm.

We used a commercially available DC motor driver which was designed based on TB6612FNG chip (Toshiba; Fig. 1b.*g*). It provides bidirectional control (clock- and counter clock-wise) using two TTL channels. To control the DC motor, the output terminal of the digital IO device (NI PCI-6601, National Instruments) was connected to the motor controller through cables (Fig. 1a).

The frame of commutator was constructed using two acrylic boards (100 × 100 mm) and steel posts (PCB supports, height of 130 mm) as shown in Fig. 1b. The boards were used for top and bottom of the frame and posts were served as vertical supports for the frame and embedded firmly at each edge of the board. The slip-ring, DC motor, and motor driver were then tightly secured on the frame using either glues or screws. Several holes for slip-ring and posts were drilled on the bottom board.

#### Third step: development of algorithm

The head position is defined by the mid-point between the LEDs. The head direction is defined by the angle composed of a reference line (e. g., y axis of an image) and the midline of animal (rostral to caudal), which is orthogonal to the axis passing both LEDs from the video image. We took advantage of the data acquisition software, Cheetah (Neuralynx). Tracking parameters were then obtained at 29.97 fps (frames per second) and streamed to processing computer through a NetCom library (Neuralynx) for online processing. Customized software was designed using Labview (National Instruments) to apply the algorithm described below.

The behavioral task consisted of a single trial repeated many times during a daily session. Across the entire session the mice spent the majority of their time near the starting position, therefore we set a virtual rectangle near the starting position in the video image (Fig. 2a). The rectangle was set empirically so that mouse could not rotate within it and could be detected when coming back to the position for reward. Under the condition, only the choice phase was needed to examine whether a mouse rotated 360°. The head direction, the relative angle from the positive Y axis of the video image, increases along the clockwise angular direction. When the mouse is near the start point, its head direction is about 180°. A single rotation of 360° through both direction (clock-wise; CW or counter-clock-wise; CCW) has a trajectory of sudden change of the angle of which absolute value is larger than a threshold value, say, 330°. Consequently, the total rotation number (TRN) is rotations of CW – CCW. For example, the TRN of +1 and −2 indicate a single rotation of CW, and two rotations of CCW direction, respectively. Zero indicates no rotation.

In summary, algorithm to detect whether a mouse rotated is as follows (Fig. 2b).

i. When the mouse occupies the starting position, wait.
ii. As soon as the mouse departs the starting position, stores a trajectory of head direction until the mouse comes into the position back.
iii. Compute rotation number (RN) from the trajectory.
  A. Count CW rotation (RNCW)
  B. Count CCW rotation (RNCCW)
  C. Total rotation number (TRN) is then TRN = RNCW - RNCCW.
iv. Generate motor command for the given TRN.

To drive the DC motor, we used pulse modulated control. We set single pulse duration as 100 ms, and changed its duty cycle. Then we generated a series of pulses, a pulse train, to drive the motor. As described above, actual disentanglement of tether is carried out when the mouse is in the start position, rewarding and/or initiating the next trial. Therefore, it is important to rotate the DC motor exactly 360°. Detailed calibration procedures are described in the next section.

#### Final step: calibration and application

There are two parameters to control the motor angles, the pulse width and the number of pulses. Therefore, by inspecting the combination of both parameters, it is possible to rotate the tether close to 360°. Initially we set the pulse width as 20 ms, that is, duty cycle of 20 %. Then pulse trains of having 20 to 30 pulses were transmitted to motor controller. We found that 26 pulses made one rotation nearly perfectly. The given pulse trains were further validated by sending it repeatedly, under simultaneous online recording of its angles. We checked the angle difference before and after motor rotation.

Finally, the designed system was applied to mouse performing behavioral tasks which lasted approximately 50 min and composed of more than 100 trials.

## Additional files

Additional file 1:
**Movie 1.** Defocusing of LED images for improvement of SNR. (MP4 229 kb) Additional file 2:
**Movie 2.** Active commutator applied to a freely behaving mouse. (MP4 520 kb)

## Abbreviations

CW: Clock-wise
CCW: Counter-clock-wise
RN: Rotation number
SNR: Signal-to-noise ratio
TRN: Total rotation number
